# A moderate reduction in irrigation and nitrogen improves water-nitrogen use efficiency, productivity, and profit under new type of drip irrigated spring wheat system

**DOI:** 10.3389/fpls.2022.1005945

**Published:** 2022-10-10

**Authors:** Wenliang Wan, Yanhui Zhao, Xiaofang Li, Jing Xu, Kaige Liu, Sihui Guan, Yaqian Chai, Hongjun Xu, Hongxin Cui, Xianjun Chen, Pei Wu, Ming Diao

**Affiliations:** ^1^ The Key Laboratory of Oasis Eco-Agriculture, Xinjiang Production and Construction Corps, Shihezi University, Shihezi, China; ^2^ College of Plant Protection, Shandong Agricultural University, Taian, China; ^3^ Crop Research Institute of Xinjiang Academy of Agricultural Sciences, Shihezi, China

**Keywords:** agronomic efficiency of nitrogen fertilizer, drip irrigation, economic profit, grain yield, spring wheat, water-use efficiency

## Abstract

Rational irrigation and nitrogen management strategies are crucial for wheat growth. However, the optimal amount of water and nitrogen for the newly developed drip irrigated spring wheat system (TR6S, one drip tube service for six rows of wheat, with a row spacing of 10 cm and an inter-block space of 25 cm, saves drip tubes and obtains higher profits) in dry and semi-arid areas remains unclear. Therefore, a field experiment was conducted with four nitrogen levels (300, 270, 240, and 0 kg ha^−1^ referred N300, N270, N240, and N0) and four irrigation levels (4500, 4200, 3900, and 3600 m^3^ ha^−1^ referred I4500, I4200, I3900, and I3600) during the 2021–2022 and 2022–2023 spring wheat seasons to analyze the effects of irrigation (I) and nitrogen (N) levels on grain yield, water-nitrogen use efficiency, profit, biomass accumulation, and nitrogen nutrient absorption status under TR6S. Compared with the traditional irrigation and nitrogen management strategy (N300–I4500, as control), lesser irrigation and nitrogen supply (I<3979 m^3^ ha^−1^ and N<273 kg ha^−1^) saved cost but led to lower grain yield, water use efficiency (WUE), agronomic efficiency of nitrogen fertilizer (AEN), and profit. However, a moderate reduction in irrigation and nitrogen supply (4500 m^3^ ha^−1^>I>3979 m^3^ ha^−1^ and 300 kg ha^−1^ >N>273 kg ha^−1^) improved grain yield, WUE, AEN, and profit. The increase in grain yield was mainly related to the rise in 1000-grain weight and kernels per spike. Although the moderate reduction in irrigation lowered soil moisture status, the dry matter pre-stored in the vegetative organs before anthesis that gets redistributed into grains during grain filling was improved. Moreover, the moderate reduction in nitrogen supply resulted in a more reasonable nitrogen nutrition index (NNI) of wheat plant, which improved flag leaf area and chlorophyll relative content (SPAD) at the anthesis stage. This also played a positive role in biomass accumulation and redistributed, yield structure optimization. Considering comprehensively yield, WUE, AEN and profit, combination of 285 kg ha^−1^ N and 4170 m^3^ ha^−1^ I was optimal irrigation and nitrogen application pattern for TR6S. This strategy can be applied to other arid and semi-arid regions.

## 1 Introduction

Shortage of fresh water resources has become a severe problem in agricultural regions worldwide (Savary et al., 2005; Si et al., 2020). Xinjiang Uygur Autonomous Region is a typical arid and semi-arid region of China with minimal freshwater resources; here, the annual rainfall is only one-tenth of evapotranspiration (Deng et al., 2006; Wan et al., 2018; Lv et al., 2019; Wan et al., 2022). Water scarcity has adversely affected the growth and production of wheat, the most important food crop in this region (Lv et al., 2019). Although drip irrigation, which uses underground pipelines to redistribute groundwater to irrigate crops, has been widely promoted in wheat production in Xinjiang (Chen et al., 2015), groundwater exploitation will eventually lead to a decline in groundwater levels, posing a threat to sustainable agriculture (Han et al., 2016; Si et al., 2020). Moreover, to achieve high crop yield, farmers often apply more fertilizers, especially nitrogen fertilizers (Si et al., 2020), which increases the cost of production and the risk of greenhouse gas emissions and groundwater pollution (Wang et al., 2016; Liang et al., 2017; Tan et al., 2017). Therefore, reasonable water and nitrogen management are important for sustainable wheat production in Xinjiang.

Optimizing water and nitrogen can improve wheat canopy structure and photosynthetic properties, promote material accumulation and redistribution, and ultimately have a positive effect on the overall yield and water-nitrogen use efficiency (Albrizio et al., 2010; Mon et al., 2016; Dar et al., 2017; Zhang et al., 2017; Si et al., 2020). In the drip irrigated spring wheat system of Xinjiang, the traditional and optimal irrigation and N application rates for high wheat yield are 4500 m^3^ ha^−1^ and 300 kg N ha^−1^, respectively (Liu et al., 2013; Jin et al., 2014; Lv et al., 2019). However, most studies are based on the traditional drip irrigated wheat planting system in Xinjiang with high cost of drip tubes (TR4; one drip tube service four rows of wheat, row spacing of 15 cm) (Liu et al., 2013; Jin et al., 2014; Lv et al., 2019). Recently, to reduce the cost of drip tubes and achieve higher economic profit, we have developed a new type of drip irrigated spring wheat system called TR6S, which serves more wheat rows with a single drip tube and uses narrow row spacing (Wan et al., 2022). This system uses one drip tube service for six rows of wheat, with a row spacing of 10 cm and an inter-block space of 25 cm (**Supplementary Figure 1**) (Wan et al., 2022). TR6S with the traditional irrigation (4500 m^3^ ha^−1^) and nitrogen (300 kg N ha^−1^) levels increased the economic profits by 3.44%–5.35% and reduced costs by 12.32% compared with TR4 (the grain yield was not significantly different with TR4) (Wan et al., 2022).

Under drip irrigation, water and fertilizer are supplied to the root areas at meager rates, which alters the distribution of water, nutrients, and roots in the soil (Li et al., 2018). This helps to promote the absorption of water and nutrients by wheat (Jha et al., 2017; Si et al., 2020). However, the horizontal transport of irrigation water mainly depends on the distance between the drip tube and the wheat row (Wang et al., 2006; Payero et al., 2008; Wang et al., 2013). Compared with TR4, TR6S not only reduced the distance between drip irrigation tube and wheat row by reduce row spacing (Wan et al., 2022), but also have higher water use efficiency (WUE) due to the significant increase of WUE at the distant rows from the drip tube [unpublished data, the WUE calculated by the differentiation of stable carbon isotopes (δ^13^C)]. From the perspective of crop physiology, crops tend to show higher WUE in response to drought [the distance between drip tube and the farthest rows of TR6S (27.5 cm) was longer than TR4 (22.5 cm)] (Abbate et al., 2004; Zhang et al., 2020). In addition, the distant rows of TR6S have margin effects in the farthest wheat rows, as exemplified by the improved grain yield and biomass of the distant rows in relation to R1 (the 1st rows adjacent to the drip tube) (Wan et al., 2022). This can also partially compensates for the reduced production due to the distance to the farther row is larger with TR6S (27.5 cm) than with the TR4 (22.5 cm). Therefore, TR6S in Xinjiang may be an effective drip irrigation system, where the irrigation and nitrogen levels may be lower than the traditional I and N application rates. Secondly, most of the research on irrigation and fertilizer on drip irrigation spring wheat were based on the plot experiment of artificial sowing in Xinjiang, and the drip tube was laid on the land surface (land surface drip irrigation system) (Liu et al., 2013; Jin et al., 2014; Lv et al., 2019). This approach differs from the current “surface shallow-buried drip irrigation system” of wheat in Xinjiang, where the drip tube is buried 2 cm under the soil surface with a seeder. The shallow-buried drip tube can effectively prevent the drip tube from being blown away by the wind after sowing (**Supplementary Figure 2**) (Wan et al., 2022). Typically, drip irrigation changes the infiltration form of soil moisture. The surface shallow-buried drip irrigation system can effectively reduce surface evaporation and runoff and plays a key role in water and fertilizer conservation (Wang et al., 2009; Yao et al., 2021). Therefore, water and nitrogen application under the surface shallow-buried drip irrigation system may be less than in the land surface drip irrigation system. Therefore, to reduce the cost and find the optimal irrigation and nitrogen strategy for TR6S, a further reduction in water and nitrogen input compared with the traditional water (4500 m^3^ ha^−1^) and nitrogen (300 kg N ha^−1^) strategies is necessary, in combination with shallow-buried drip irrigation.

Therefore, the present study used the new drip irrigated spring wheat system of TR6S, combined with shallow-buried drip irrigation. We explored the effects of different irrigation and nitrogen application rates, lower than the traditional levels, on spring wheat in two field trials (2021 and 2022). The objectives of this study were: (i) to compare performances of grain yield, water-nitrogen use efficiency and economic profits under different irrigation and nitrogen application rates; (ii) to reveal the variations in yield components, biomass accumulation and redistributed, nitrogen nutrition index of plants to explain the performances of grain yield, water-nitrogen use efficiency and economic profits under different irrigation and nitrogen application rates; and (iii) to determine an optimal irrigation and nitrogen fertilizer combinative application pattern for new drip irrigated spring wheat system in Xinjiang.

## 2 Materials and methods

### 2.1 Experimental design

The field experiment was conducted during two growing seasons of spring wheat (2021 and 2022) at the experimental farm of Shihezi University of Xinjiang Uyghur Autonomous Region, China (**Supplementary Figure 3A**). The daily maximum and minimum air temperatures and the daily precipitation over the experimental period are shown in **Supplementary Figure 3B**. The wheat cultivar XC 39 (*Triticum aestivum* L. Xinchun 39) widely grown in Xinjiang was used in this study. The wheat seeds were sown on 6 April 2021 and 8 April 2022. The soil classified as light loam with a pH of around 7.6 has a water capacity of 26.9%, a total porosity of 45.5%, and a bulk density of 1.23 g cm^−3^. The content of soil organic matter, available N, Olsen-P, and available potassium before sowing were 11.4 g kg^−1^, 42.1 mg kg^−1^, 13.8 mg kg^−1^, and 295 mg kg^−1^, respectively.

**Figure 1:**
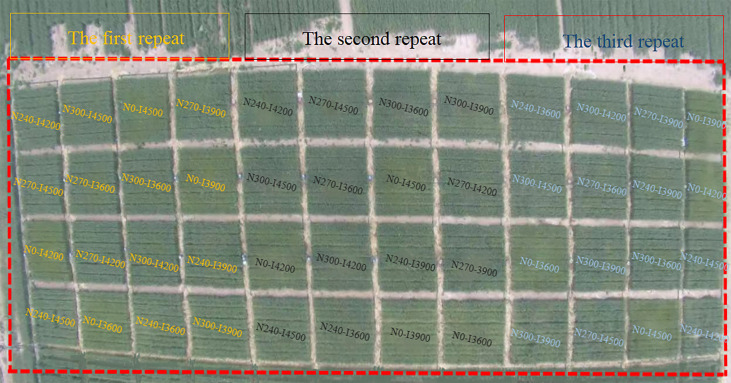
The experimental plot layout (16 treatments, three replicates, a total of 48 experimental plots). *Notes:* I4500, I4200, I3900, and I3600 indicate 4500, 4200, 3900, and 3600 m^3^ ha^−1^ irrigation levels, respectively. N300, N270, N240, and N0 indicate 300, 270, 240, and 0 kg ha^−1^ nitrogen levels, respectively.

The new drip irrigation system TR6S was selected for the spring wheat planting pattern in the two experimental years. The experiment was conducted in a randomized block design with two factors, including irrigation levels (4500, 4200, 3900, and 3600 m^3^ ha^−1^; I4500, I4200, I3900, and I3600) and nitrogen levels (300, 270, 240, and 0 kg ha^−1^; N300, N270, N240, and N0). The traditional nitrogen and irrigation levels (N300, I4500) of Xinjiang were used as control (CK). Thus, the experiment was performed using 16 treatments with three replicates per treatment, making a total of 48 experimental plots (**Figure 1**); the area of each experimental plot was 30 m^2^ (5 m × 6 m). The planting density was used as per the previous studies and locally recommended protocols (Liu et al., 2013; Jin et al., 2014). Precision wheat seeder (JB/T 6274.1-2013, 2BFX-12, China) was used for simultaneous sowing and laying drip tubes; the shallow drip tubes were buried at a depth of 1–2 cm from the surface soil (**Supplementary Figure 2**). The irrigation and nitrogen strategy (the source of nitrogen is urea (CH_4_N_2_O) with 46% nitrogen content) in each growth period was carried out following previous reports (Liu et al., 2013; Jin et al., 2014; Lv et al., 2019) (**Table 1**). In addition, a dose of 105 kg ha^−1^ P_2_O_5_ and K_2_O was applied to the soil before sowing.

**Table 1:** Irrigation and nitrogen application ratios during different growth periods of wheat.

Growth stage	Three-leaf stage	Jointing stage	Booting stage	Anthesis	Early milk stage	Late milk stage
Irrigation (m^3^ ha^-1^)	20%	20%	20%	15%	15%	10%
Nitrogen (kg ha^-1^)	32%	32%	16%	12%	8%	−

### 2.2 Sampling and measurement

#### 2.2.1 Biomass, leaf area, and SPAD

Twenty spring wheat plants were harvested at both anthesis and maturity. The plants were divided into leaves, stems, sheathes, and spikes at anthesis and into leaves, stems, sheathes, chaffs, and grains at maturity. All samples were dried at 85°C in an oven to a constant weight to determine total dry matter accumulation (TAD, kg m^−2^), dry matter accumulation before anthesis (DA−B, biomass at anthesis, kg m^−2^), and dry matter accumulation after anthesis (DA−A, difference in biomass between maturity and anthesis, kg m^−2^). The amount of dry matter accumulated before anthesis and redistribution into grains after anthesis (DAR, kg m^−2^), the redistribution rate of DAR to grain (DAR−R, %), the contribution rate of DAR to grain (DAR−C, %), the ratio of DA−A to TAD (PATD, %), and the harvest index (HI) were calculated using the following Eqs. (1)–(5) (Wheeler et al., 1996; Plaut et al., 2004; Lv et al., 2019; Wan et al., 2022):


Eq. (1) $$DAR(gm^{-2})=dry\;matter\;accumulation\;at\;anthesis–dry\;matter\;accumulation\;of\;vegetation\;at\;maturity$$

Eq. (2) DAR−R(%)=DAR/Dry matter accumulation at anthesis×100%

Eq. (3) DAR−C(%)=DAR/Grain weight at maturity×100%

Eq. (4) PATD(%)=DA−A/Dry matter accumulation at maturity×100%

Eq. (5) HI=Grain weight/Dry matter accumulation at maturity

In addition, twenty wheat plants were randomly selected from different treatments during the anthesis stage to measure SPAD using a Chlorophyll meter (SPAD−502PLUS, Konica Minolta Inc., Japan). Another thirty wheat plants were randomly selected from different treatments during the anthesis stage to measure the flag leaf area according to the method of Huang et al. (2020).

#### 2.2.2 Soil water content and water-use efficiency (WUE)

The soil moisture content was determined using soil moisture determination tubes (Profile Probe type, Delta−T Devices, Cambridge, England), according to the method of Lv et al. (2019). The soil moisture was measured to a depth of 100 cm (0–20, 20–40, 40–60, and 60–100 cm). In 2021 and 2022, soil water content was measured at four growth stages of wheat: trifoliate stage, jointing stage, anthesis stage, and filling stage. In addition, the evapotranspiration (ETc) of wheat was calculated by measuring the change in the water content in the soil profile before sowing and after harvest as follows (Rana and Katerji, 2000; Kresović et al., 2016; Lv et al., 2019):


Eq (6) $${\text{ET}}_{\text{c}}{\text{=I+P+C}}_{\text{r}}-{\text{R}}_{\text{f}}-{\text{D}}_{\text{p}}\pm \text{ΔS}$$

Where ETc is the evapotranspiration (mm) of the whole growth period of wheat; I and P represent the irrigation amount (mm) and rainfall (mm) during the whole growth period; C_r_, D_p_, and R_f_ indicate capillary rise (mm); percolation (mm), and runoff (mm) in the field; ΔS represents soil moisture change (mm) before sowing and after harvest. The measured values of C_r_, D_p_, and R_f_ were zero during the two growing seasons. The WUE (kg mm^-1^) was calculated by dividing grain yield (kg) by ET_c_ (mm) (Si et al., 2020).

#### 2.2.3 Nitrogen nutrition index (NNI)

The nitrogen nutrient index (NNI) was calculated based on the critical nitrogen concentration index function of spring wheat under drip irrigation in northern Xinjiang proposed by Peng et al. (2015). NNI was used to quantify the nitrogen status of the crops. NNI less than 1 indicates insufficient nitrogen; NNI equal to 1 indicates that crop nitrogen absorption is appropriate; NNI greater than 1 indicates excessive nitrogen supply (Peng et al., 2015; Wan et al., 2018). The NNI was calculated using Eq. (7) (Peng et al., 2015; Wan et al., 2018):


Eq. (7) NNI=Nt/Nc

Where Nt is the measured value of aboveground biomass nitrogen concentration. Nc represents the nitrogen concentration (%) at the same aboveground biomass obtained according to the critical nitrogen concentration power function of drip irrigated spring wheat in Xinjiang (N_C_ = 5.03×W^−0.38^, R^2^ = 0.95). W represents dry weight of the aboveground biomass. Ten wheat plants were taken from each experimental area every 8 days after wheat emergence, and the samples were initially kept for 0.5 h in an oven at 105 °C and dried at 75 °C until a constant weight. Dried plant samples were pulverized with a small pulverizer and sieved. Nitrogen content (%) in the powder was determined using a Buchi K−375 automatic Kjeldahl nitrogen analyzer (BUCHI Labortechnik AG, Switzerland) at the Analysis and Testing Center of Shihezi University.

#### 2.2.4 Grain yield and agronomic efficiency of nitrogen fertilizer (AEN)

After entering the mature stage, the spike number of each plot was investigated. Thirty plants were selected for indoor test, and indexes such as kernels per spike and 1000-grain weight were mainly evaluated. Then, at the mature stage, 1 m^2^ (1 m×1 m) of wheat was harvested for yield measurement (kg ha^−1^) in each experimental plot. The agronomic efficiency of nitrogen fertilizer (AEN) was calculated with Eq. (8) (Hu et al., 2018):


Eq. (8) $$AEN=grain\;yield\;with\;nitrogen\;treatment(kg\;ha^{-1})-grain\;yield\;with\;no\;nitrogen(kg\;ha^{-1})/the\;amount\;of\;nitrogen\;fertilizer\;applied(kg\;ha^{-1})$$

#### 2.2.5 Economic profit

Economic profit was calculated using Eq. (9):


Eq. (9) $$\begin{array}{l} Profit(US\$\;ha^{-1})=grain\;yield(kg\;ha^{-1})\times wheat\;price(US\$\;kg^{-1})+wheat\;straw(US\$\;kg^{-1})-irrigation\\ water\;cost(US\$\;ha^{-1})\;fertilizer\;cost(US\$\;ha^{-1})-pesticide\;cost(US\$\;ha^{-1})-seed\;cost(US\$\;ha^{-1})-drip\\ irrigation\;equipment\;cost(US\$\;ha^{-1})-sowing\;machinery\;and\;labor\;costs\;(US\$\;ha^{-1}) \end{array}$$

### 2.3 Statistical analysis

All data were analyzed using one-way analysis of variance (ANOVA) in the Statistical Product and Service Solutions software (SPSS Inc., Chicago, IL, USA) to compare the differences between the different irrigation and nitrogen application rates. The charts were generated with Origin 9 software (Systat Software, Inc., San Jose, California, USA).

## 3 Results and discussion

### 3.1 Moderate reduction in irrigation and nitrogen effectively increases yield, WUE, AEN, and profit under the new drip irrigated spring wheat system

Under the new drip irrigated spring wheat system (TR6S) with traditional irrigation and nitrogen levels (N300-I4500) (CK) in Xinjiang, the grain yield was 8015 kg ha^−1^ in 2021 and 8190 kg ha^−1^ in 2022 (**Table 2**). Meanwhile, when the nitrogen and irrigation levels were reduced to 270 kg ha^−1^ and 4200 m^3^ ha^-1^ (N270−I4200), the grain yield significantly increased compared with N300−I4500. The increase in grain yield was 8%–8.3% for N270−I4200. In both years, N270−I4200 showed the highest grain yield. The N300-I4200 and N270-I4500 treatments also increased grain yield compared with N300-I4500; however, the yield was lower than that of N270−I4200. The grain yield increased by 3.0%–8.0% for N300−I4200 (significant difference in yield increase) and 0.6%–0.7% for N270−I4500 (no significant difference in yield increase). However, a further reduction in water and nitrogen supply resulted in a decline in grain yield.

**Table 2:** Grain yield, water-use efficiency (WUE), agronomic efficiency of nitrogen fertilizer (AEN), total cost, and economic profit of wheat grown under different treatments in 2021 and 2022.

Year & Treatment			Yield		WUE		AEN		Total cost		Profit	
		kg ha−1	DTCK (%)	kg ha−1 mm−1	DTCK (%)		DTCK (%)	US$ ha−1	DTCK (%)	US$ ha−1	DTCK (%)
**2021**	I4500	8015c	—	13.5c	—	8.7e	—	865.9	—	2225d	—
	I4200	8259b	3.0	15.3a	13.1	12.0b	37.8	853.9	-1.4	2491b	12.0
N300	I3900	7729d	-3.6	14.7b	9.1	9.5d	8.5	841.9	-2.8	2173e	-2.4
	I3600	6805f	-15.1	13.6c	1.0	7.0f	-20.3	829.9	-4.2	1824i	-18.0
N270	I4500	8064c	0.6	13.7c	1.5	10.0cd	14.8	850.3	-1.8	2275c	2.3
	I4200	8652a	8.0	15.4a	14.0	13.7a	56.3	838.3	-3.2	2536a	14.0
	I3900	7731d	-3.5	14.7d	9.1	10.5c	20.6	826.3	-4.6	2189de	-1.6
	I3600	6506g	-18.8	13.0d	-3.4	6.6f	-24.1	814.3	-6.0	1723j	-22.6
N240	I4500	7273e	-9.3	12.4e	-7.9	8.2e	-6.2	834.8	-3.6	2002g	-10.0
	I4200	7355e	-8.2	13.1d	-3.1	10.0d	14.0	822.8	-5.0	2046f	-8.1
	I3900	6921f	-13.6	13.2d	-2.3	8.5e	-2.9	810.8	-6.4	1889h	-15.1
	I3600	5791h	-27.7	11.6f	-14.0	4.5g	-48.7	798.8	-7.8	1460k	-34.4
N0	I4500	5305i	-33.8	9.1h	-32.8	—	—	710.3	-18.0	1359l	-38.9
	I4200	4964j	-38.1	8.8i	-34.6	—	—	698.3	-19.4	1238m	-44.4
	I3900	4884jk	-39.1	9.3g	-31.1	—	—	686.3	-20.7	1218m	-45.2
	I3600	4716k	-41.2	9.5g	-30.0	—	—	674.3	-22.1	1165n	-47.6
**2022**	I4500	8190b	—	13.9c	—	8.8e	—	865.9	—	2328b	—
	I4200	8843a	8.0	15.8a	13.3	12.1b	37.7	853.9	-1.4	2595a	11.5
N300	I3900	7969c	-2.5	15.0b	7.7	9.5d	7.6	841.9	-2.8	2266c	-2.7
	I3600	7033f	-14.5	13.9c	0.1	6.9f	-21.5	829.9	-4.2	1913g	-17.8
N270	I4500	8239b	0.7	13.9c	0.2	10.0cd	13.2	850.3	-1.8	2363b	1.5
	I4200	8874a	8.3	15.8a	13.9	13.6a	54.3	838.3	-3.2	2623a	12.7
	I3900	7962c	-2.6	15.0b	8.0	10.5c	19.3	826.3	-4.6	2279c	-2.1
	I3600	6770g	-17.8	13.5d	-2.6	6.7f	-23.8	814.3	-6.0	1826h	-21.6
N240	I4500	7543d	-9.6	12.8e	-8.1	8.3e	-5.6	834.8	-3.6	2107e	-9.5
	I4200	7625d	-7.5	13.6d	-2.1	10.1c	14.5	822.8	-5.0	2151d	-7.6
	I3900	7189e	-13.1	13.6d	-2.5	8.6e	-2.3	810.8	-6.4	1993f	-14.4
	I3600	6048h	-29.8	12.1f	-13.0	4.5g	-48.4	798.8	-7.8	1560i	-33.0
N0	I4500	5547i	-43.7	9.4i	-32.7	—	—	710.3	-18.0	1453j	-37.6
	I4200	5204j	-53.8	9.2i	-33.8	—	—	698.3	-19.4	1331k	-42.8
	I3900	5124j	-58.9	9.6h	-30.7	—	—	686.3	-20.7	1312k	-43.6
	I3600	4957k	-63.1	9.8g	-29.2	—	—	674.3	-22.1	1259l	-45.9
F Year		330.124 **		169.871 **		3.465				330.124 **	
F Irrigation		94.641 **		31.655 **		317.657 **				84.799 **	
F Nitrogen		438.230 **		414.832 **		112.826 **				309.284 **	

Traditional water and nitrogen levels (N300-I4500) used as control (CK), DT_CK_ indicates the percentage difference between CK and other treatments. The lowercase letters refer to significant differences among the treatments at the 0.05 level. F_Year_, F_Irrigation_, and F_Nitrogen_ refer to the F-value between years, irrigation levels, and nitrogen levels, respectively. ** indicate significant differences at 0.05 and 0.01 levels, respectively. The price of wheat grains, single bypass, drip tubes, and wheat straw was 0.38 US$ kg−1, 0.042 US$, 0.027 US$ m−1 and 120 US$ ha−1, respectively. Other costs included water consumption (180 US$ ha^−1^), seed (165 US$ ha^−1^), fertilizer and pesticide (156 US$ ha^−1^) costs, and sowing machinery and labor costs (120 US$ ha^−1^).

Furthermore, a moderate reduction in water and nitrogen supply compared with traditional water and nitrogen levels (N300−I4500) increased the WUE and AEN (**Table 2**). Among them, N270−I4200 maintained the highest WUE and AEN in both years. Compared with N300-I4500, N270-I4200 increased the WUE and AEN by 13.9%–14.0% and 53.4%–56.3%, respectively. However, with a more considerable reduction in the nitrogen and irrigation levels, the WUE and AEN significantly decreased. The decline in water and nitrogen levels resulted in lower total costs (**Table 2**). Finally, compared with the traditional water and nitrogen treatment (N300−I4500), the three experimental treatments (N270−-I4200, N300−I4200, and N270−I4500) significantly improved economic profits in both years; the treatment N270−I4200 reduced the cost by 3.2%, increased the yield by 8.0%–8.3%, and obtained the highest profit.

**Figure 2:**
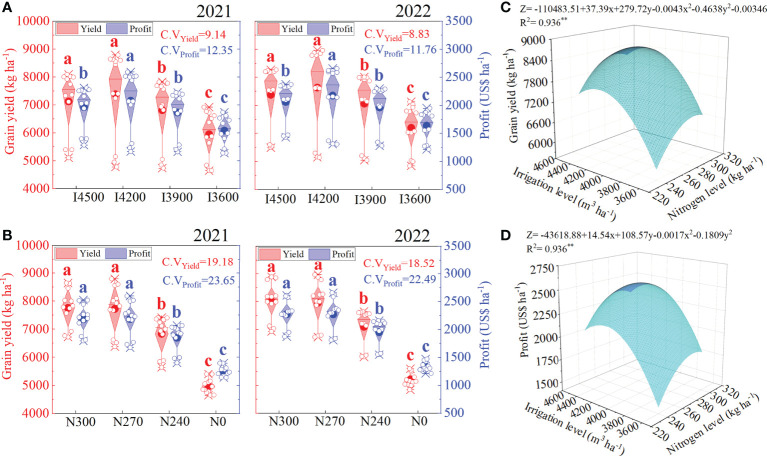
Effects of irrigation and nitrogen levels on wheat yield and economic benefits. **(A, B)** represent the effect of irrigation and nitrogen on yield and profit, respectively; **(C, D)** represents the combined effect of irrigation and nitrogen on yield and profit, respectively. I4500, I4200, I3900, and I3600 indicate 4500, 4200, 3900, and 3600 m^3^ ha^−1^ irrigation levels, respectively. N300, N270, N240, and N0 indicate 300, 270, 240, and 0 kg ha^−1^ nitrogen levels, respectively. C.V_Yield_ and C.V_Profit_ indicate the coefficient of variation in grain yield and profit among the irrigation and nitrogen levels, respectively. The lowercase letters refer to significant differences among the treatments at the 0.05 level.

Thus, the yield and profit first increased and then declined with a decrease in the irrigation and nitrogen levels in both years (**Figures 2A, B**). These observations indicate that under the new drip irrigated system of TR6S, a moderate reduction in the irrigation and nitrogen levels compared with the traditional irrigation amount and nitrogen fertilizer application amount resulted in the more satisfactory grain yield and profit. In contrast, an excessive reduction resulted in a significant decline in grain yield and economic gain. Previous studies on the yield performance of the traditional drip irrigated wheat planting system of Xinjiang showed that too much or too little irrigation did not result in the highest yield; yield and evapotranspiration showed a quadratic relationship with an initial increase and then a decrease (Chen et al., 2015). Specifically, moderate irrigation increased nitrogen accumulation in wheat’s vegetative and reproductive organs, which benefitted grain filling (Hu et al., 2018). Although the nitrogen content in the wheat vegetative organs increased with the increase in nitrogen application rate, nitrogen transfer to the grains first increased and then decreased, limiting the increase in yield (Peng et al., 2015; Hu et al., 2018). Liu et al. (2016) also found that the nitrogen application rate significantly influenced yield, but it first increased and then decreased with the increase in nitrogen application rate. A very high or low nitrogen application rate did not result in a satisfactory yield, consistent with the results of the present experiment. However, compared with the traditional irrigation (4500 m^3^ ha^−1^) and nitrogen application (300 kg ha^−1^) levels, when the irrigation and nitrogen application levels were reduced to 4200 m^3^ ha^−1^ and 270 kg ha^−1^, the difference of yield and profit increase was not significant (only except the profit of I4200 was significantly higher than I4500 in 2022). This indicates that the effects of either nitrogen or irrigation treatment on the yield and profits of TR6S planting systems were all limited. Therefore, the combined effect of irrigation and nitrogen on yield and profit should be taken seriously. Data fitting revealed that 286 kg ha^−1^ nitrogen supply and 4239 m^3^ ha^−1^ irrigation water resulted in the highest grain yield (**Figures 2C, D**); however, the increase in nitrogen and irrigation supply at lower levels positively influenced grain yield. When the nitrogen application rate was greater than 286 kg ha^−1^, the rise in yield mainly depended on the increase in irrigation level; here, the nitrogen supply limited the increase in yield. When the irrigation amount was greater than 4239 m^3^ ha^−1^, the increase in yield was mainly dependent on the increase in nitrogen level; here, the irrigation amount limited the increase in yield. Moreover, obtaining the highest yields does not necessarily mean the highest economic returns, as costs vary with nitrogen and irrigation levels (**Table 2**). The data fitting showed maximum economic profits at 285 kg ha^−1^ nitrogen supply and 4170 m^3^ ha^−1^ irrigation amount. In addition, the variation coefficient of yield and profit under various nitrogen levels (C.V_Yield_ and C.V_Profit_) (18.52–23.65) was significantly higher than that of yield and profit under different irrigation levels (8.83–12.35), which indicated that a change in nitrogen supply had a greater impact on yield and profit than that in irrigation level. Therefore, we should be more cautious while adjusting wheat’s nitrogen fertilizer application rate.

It is worth noting that rainfall is an important reference value in agricultural production (Wan et al., 2018), and the fluctuation of rainfall between years will inevitably affect the change of optimal water and fertilizer supply in crops. However, through the rainfall data for nearly eight years, the local rainfall is stable and less than 200 mm (Wan et al., 2018; Lv et al., 2019; Wan et al., 2022). Therefore, the optimal water nitrogen levels recommended for this trial are stable of TR6S in Xinjiang.

### 3.2 The increase of grain yield was mainly due to the increase of 1000-grain weight and kernels per spike

**Figure 3:**
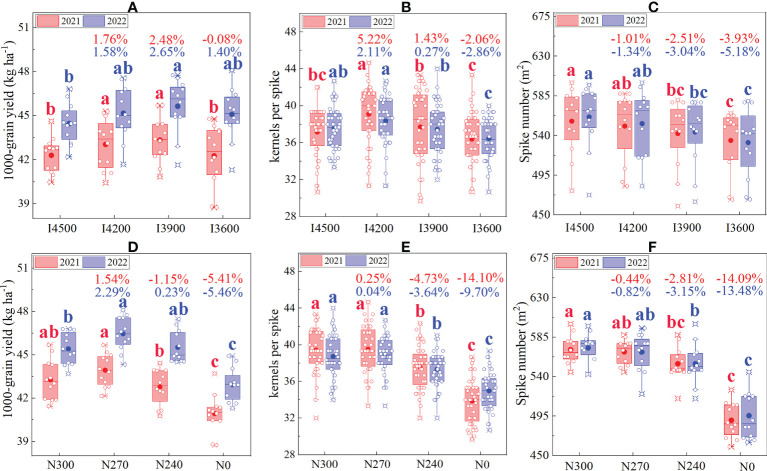
Effects of irrigation and nitrogen levels on wheat yield components (1000-grain weight, kernels per spike, and spike number). **(A–C)** represent the performance of 1000-grain weight, kernels per spike and spike number under different irrigation levels; **(D–F)** represent the performance of 1000-grain weight, kernels per spike and spike number under different nitrogen levels, respectively. I4500, I4200, I3900, and I3600 indicate 4500, 4200, 3900, and 3600 m^3^ ha^−1^ irrigation levels, respectively. N300, N270, N240, and N0 indicate 300, 270, 240, and 0 kg ha^−1^ nitrogen levels, respectively. The numbers in the columns indicate the percentage change in yield components (1000-grain weight, kernels per ear, and spike number) under a given treatment compared with conventional irrigation (I4500) and nitrogen levels (N300). The lowercase letters refer to significant differences among the treatments at the 0.05 level.

Yield is a comprehensive response of various components, such as 1000-grain weight, kernels per spike, and spike number; any change in these components will eventually affect yield (Chen et al., 2015). In this experiment, when the irrigation level continuously declined from 4500 m^3^ ha^−1^ (I4500), the spike number showed a decline in both years (**Figure 3C**). Compared with I4500, the I4200, I3900, and I3600 treatments reduced the spike number by 1.01%–1.34% (the difference was not significant), 2.51%–3.04% (the difference was significant), and 3.93%–5.18% (the difference was significant), respectively. When the nitrogen level declined from 300 kg ha^−1^ (N300), the spike number showed a continuous decline (**Figure 3F**). Compared with N300, the N270, N240, and N0 treatments reduced the spike number by 0.44%–0.82% (the difference was not significant), 2.81%–3.15% (the difference was significant), and 13.48%–14.09% (the difference was significant), respectively. The decline in both irrigation and nitrogen levels led to a decrease in spike number, consistent with previous studies (Chen et al., 2015; Hu et al., 2018; Lv et al., 2019). Generally, the spike number increases mainly due to the production of more effective tillers at the seedling stage. The formation of effective tillers is often positively correlated with adequate water and nitrogen supply (Wardlaw, 2002; Hu et al., 2018). Interestingly, with the decrease in irrigation and nitrogen levels from 4500 m^3^ ha^−1^ (I4500) and 300 kg ha^−1^ (N300), respectively, in most case, the 1000-grain weight and spike number first significantly increased and then decreased (**Figures 3A, B, D, E**). Due to the grain grouting process often requires a more adequate supply of water and fertilize, and low irrigation and nitrogen can inhibit grain filling, resulting in lighter 1000-grain weight and less spike number (Chen et al., 2015). This well explains the performance of the yield structure under low irrigation and nitrogen treatments in our experiment. In addition, high water and nitrogen environments often inhibit crop growth, especially leaf area index (LAI), which reduces photosynthetic rate, reduces the efficiency of dry matter accumulation and transport, and ultimately is not conducive to the formation of yield structure (Si et al., 2020). This is consistent with the findings of this trial study.

In conclusion, a moderate reduction in water and nitrogen supply (N270–I4200) slightly reduced the spike number (-0.44%–1.32%) but significantly improved the 1000-grain weight (1.54%–2.29%) and kernels per spike (0.04%–5.22%). The variation in 1000-grain weight and kernels per spike with water and nitrogen supply showed a linear relationship with the variation in yield (**Figures 2**, **3**). Therefore, under the new drip irrigated wheat planting system of Xinjiang, an increase in yield after the moderate reduction in water and nitrogen supply was mainly attributed to the rise in 1000-grain weight and kernels per spike.

### 3.3 Biomass accumulation and redistribution play a positive role in optimizing yield components

**Figure 4:**
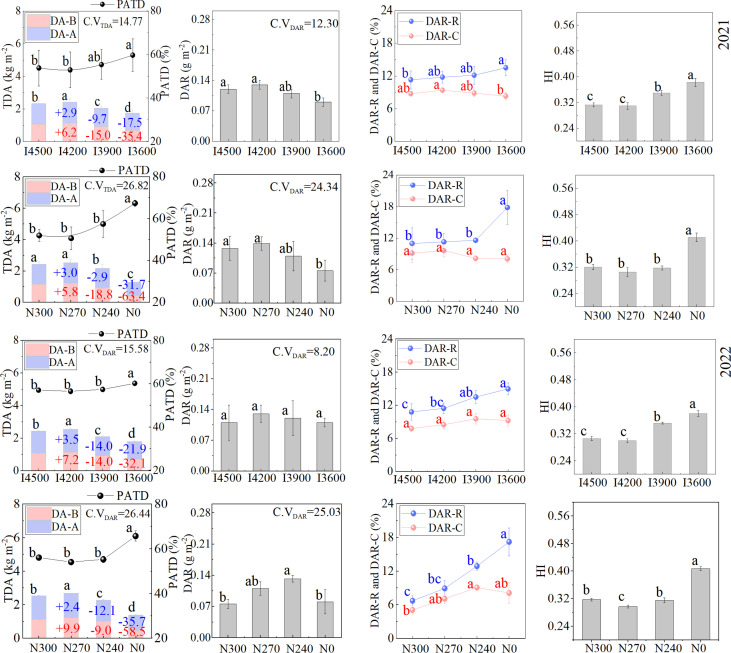
Effects of irrigation and nitrogen levels on biomass accumulation and redistribution in wheat. I4500, I4200, I3900, and I3600 indicate 4500, 4200, 3900, and 3600 m^3^ ha^−1^ irrigation levels, respectively. N300, N270, N240, and N0 indicate 300, 270, 240, and 0 kg ha^−1^ nitrogen levels, respectively. TDA: the total dry matter accumulation at maturity, DA−B: dry matter accumulation before anthesis, DA−A: dry matter accumulation after anthesis, PATD: the ratio of DA−A to TDA, DAR: DA−B redistributed into grains after anthesis, DAR−R: the rate of DAR to grains, DAR−C: the contribution of DAR to grains, HI: the harvest index. C.V_TDA_ and C.V_DAR_ indicate the coefficient of variation in TDA and DAR among the irrigation and nitrogen levels, respectively. The numbers in the red and blue columns indicate the percentage change in DA−B and DA−A under a given treatment compared with the traditional irrigation (I4500) and nitrogen levels (N300). The lowercase letters refer to significant differences among the various irrigation and nitrogen levels at the 0.05 level.

The formation of wheat grains is mainly due to the accumulation of photosynthetic assimilates; therefore, a change in yield components depends on the accumulation and redistribution of dry matter (Moll et al., 1994; Liu et al., 2016; Lv et al., 2019). In this experiment, when the irrigation level and nitrogen level continued to decline, the total dry matter accumulation (TDA) at maturity significantly increased under I4200 and N270 (no significant difference in the rise of N270 in 2021); it then declined (**Figure 4**). In addition, the translocation of dry matter accumulated before anthesis to grains after anthesis (DAR) also first increased and then decreased with the decline in water and nitrogen supply. In most cases, DAR was the best under I4200 and N270 (except, the DAR in 2022 was lower than N240 at N270). More biomass accumulation and redistribution often optimizes yield components and results in high crop yield (Gallagher et al., 1976; Yang et al., 2001; Wang et al., 2014; Wang et al., 2015). Thus, the increase in TDA and DAR after moderate reduction in irrigation and nitrogen supply improved the 1000-grain weight, kernels per spike, and overall yield (**Figures 2**–**4**). In addition, the variation in dry matter accumulation before anthesis (DA–B) with irrigation and nitrogen levels was generally greater than that in dry matter accumulation after anthesis (DA–A) (**Figure 4**). This indicates that DA–B is more sensitive to the changes in irrigation and nitrogen. However, DA–A generally accounted for more than 50% of TDA (**Figure 4**), which indicates that DA–A is more critical to TDA and yield improvement. This observation is consistent with the previous reports (Panda et al., 2003; Wan et al., 2022).

Notably, the variation coefficient of TDA under various irrigation and nitrogen levels (14.77–26.82) was higher than that of yield (8.83–12.35) (**Figures 2**, **4**), which indicates that the reduction in irrigation amount and nitrogen fertilizer had a greater impact on TDA than yield. Therefore, yield may have been somewhat compensated under water and nitrogen stress. Generally, grain formation is closely related to the accumulation and redistribution of photosynthetic assimilates (Moll et al., 1994; Liu et al., 2016; Lv et al., 2019) so, the compensation of yield might be related to biomass accumulation and redistribution. In this experiment, with the decline in irrigation and nitrogen levels, the proportion of dry matter accumulation after anthesis to total biomass (PATD) showed a gradual increase (**Figure 4**). In addition, the DAR first increased and then declined with the decline in water and nitrogen; however, the variation coefficient of DAR (8.20–25.03) was significantly lower than that of TDA (14.77-26.82), which indicates an alleviation in DAR decline with the decrease in irrigation and nitrogen supply. Moreover, when irrigation and nitrogen supply continued to decline, the translocation rate of DAR to grains (DAR–R) and the contribution rate of DAR to grains (DAR–C) increased (**Figure 4**). Subsequently, the harvest index (HI) showed an increase (**Figure 4**). Thus, the variations in PATD, DAR, DAR–R, DAR–C, and HI indicated that yield compensation was mainly due to the redistribution of dry matter pre-anthesis, consistent with our previous reports (Wan et al., 2022). This mechanism is beneficial to wheat yield formation under adversity. However, since biomass (TDA) is the most critical factor limiting yield (Chen et al., 2015), so even the continuous decline in water and fertilizer supply can effectively increase PATD, DAR–R and DAR–C to compensate for yield losses, the productivity will also decline. In conclusion, moderate reduction of water and nitrogen supply was beneficial to the improvement of TDA and DAR, and improved PATD, DAR–R, DAR–C and HI, and ultimately played an important role in the optimization of 1000-grain weight and kernels per spike.

### 3.4 Optimization of biomass accumulation and redistribution is related to the principle of deficit irrigation

**Figure 5:**
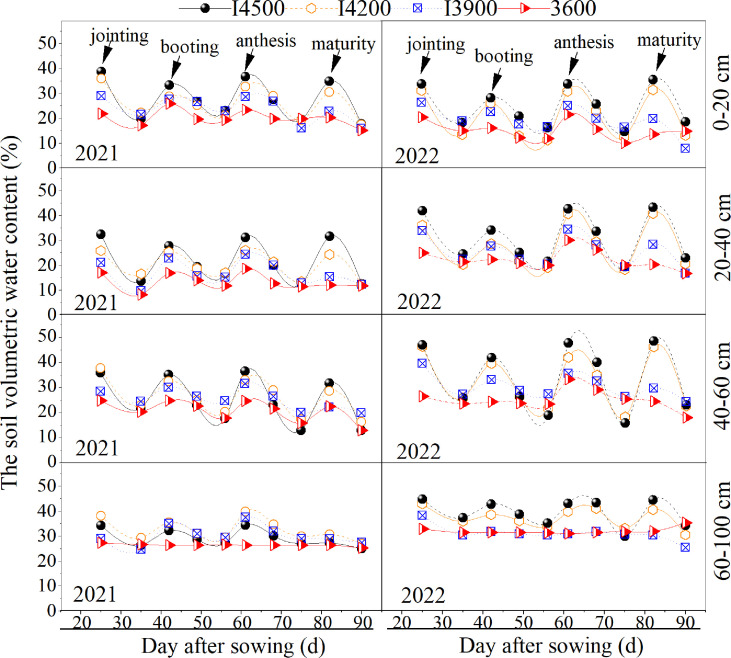
Volumetric water content in the soil during the various wheat growth stages. I4500, I4200, I3900, and I3600 indicate 4500, 4200, 3900, and 3600 m^3^ ha^−1^ irrigation levels, respectively.

Water is crucial for forming wheat biomass (Panda et al., 2003). As shown in **Figure 5**, the water content of the 0–100 cm soil layer was the highest just after irrigation, at all irrigation levels (I4500, I4200, I3900, and I3600). With time, the soil water content under each irrigation level gradually declined. After the next irrigation, the soil water content returned to almost the same level as that after the previous irrigation. It can be seen that the drip irrigation wheat continuously experiences the process of “water deficit-rewatering” with the occurrence of irrigation during the whole growth period under different irrigation levels. This well simulated the scene of deficit irrigation (Chai et al., 2016). In general, water deficit could promote the translocation rate and contribution rate of pre-anthesis stored dry matter from vegetative organs to grains (Gallagher et al., 1976; Yang et al., 2001; Wang et al., 2014; Wang et al., 2015). In our previous study, we found that that the soil water content about 37.5 cm away from the drip tube can lead to a significant reduction in wheat production (Lv et al., 2019). This indicates that wheat suffered drought stress at the water content level of this position. The volumetric moisture content of soil of this position was about 20% (0–20 cm soil layer), 25% (20–40 soil layer), 30% (40–60 cm soil layer) and 30% (60–100 cm soil layer), respectively (Lv et al., 2019). We use this soil water content as a reference (the soil water content about 37.5 cm away from the drip tube). Then, in most case, we found that the soil volume moisture content of I3900 and I3600 were lower than this reference value. This indicates that the wheat under I3900 and I3600 suffered from a relatively severe drought during the grow period. Furthermore, the volumetric water content of I4200 fluctuates around this reference value, and thus suffers from much less drought stress. Therefore, although wheat plants were subjected to deficit irrigation at all irrigation levels, the degree of deficit irrigation was different. I4200 optimized the dry matter accumulation and redistribution and ultimately improved the productivity compared with I4500 under moderately reduced soil moisture (**Figures 2**, **4**). I3900 and I3600 also transformed dry matter accumulation and redistribution; however, the productivity was not high (**Figures 2**, **4**) because dry matter accumulation was more sensitive to irrigation reduction, especially at pre-anthesis (Panda et al., 2003; Lv et al., 2019; Wan et al., 2022). The low soil moisture status of I3900 and I3600 during the whole growth period led to less biomass (**Figure 5**). Since biomass is a crucial factor in determining yield (Chen et al., 2015), so low water supply (I3900 and I3600) led to lower productivity.

### 3.5 Appropriate nitrogen nutrition increases leaf area and SPAD and also plays an important role in optimizing biomass accumulation and redistribution

**Figure 6:**
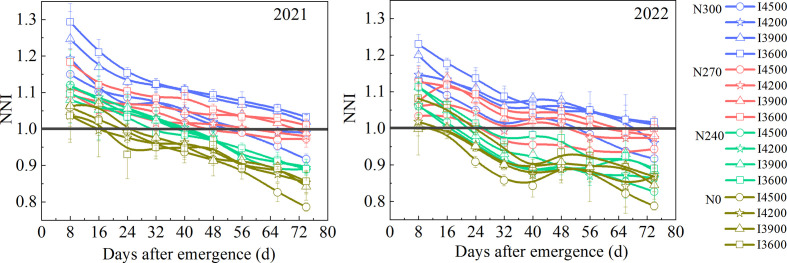
Variations in nitrogen nutrition index (NNI) of wheat plants under different treatments. I4500, I4200, I3900, and I3600 indicate 4500, 4200, 3900, and 3600 m^3^ ha^−1^ irrigation levels, respectively. N300, N270, N240, and N0 indicate 300, 270, 240, and 0 kg ha^−1^ nitrogen levels, respectively.

Nitrogen Nutrition Index (NNI) is based on crop critical nitrogen concentration, which has physiological significance and can quantitatively reflect the nitrogen nutritional status in crops (Peng et al., 2015; Liu et al., 2016; Wan et al., 2018). Guo et al. (2004) studied the effect of nitrogen distribution on the yield of drip irrigated wheat and found that the application of nitrogen fertilizer at the jointing stage significantly increased yield. In this experiment, from 16 to 32 days after wheat emergence (two leaves stage and jointing stage), the decline in NNI in each treatment was significant (**Figure 6**), which indicates high nitrogen demand during this period, consistent with previous reports (Guo et al., 2004; Hu et al., 2018). Generally, NNI>1 indicates excess nitrogen, which demands nitrogen reduction; NNI=1 is good nitrogen nutrition status; NNI<1 indicates a lack of nitrogen nutrition, which requires nitrogen fertilizer application (Peng et al., 2015; Wan et al., 2018). In this experiment, within 16 days after wheat emergence, the NNI of each treatment was greater than 1, especially the N0 treatment (N0–I4500, N0–I4200, N0–I3900, N0–I3600), indicating a soil N sufficient to meet wheat demand before jointing stage. However, beyond 16 days, the NNI of each N0 treatment declined gradually and attained a level of less than 1. N0 treatment showed insufficient nitrogen absorption after 16 days of wheat emergence. The NNI of N240 treatments (N240–I4500, N240–I4200, N240–I3900, N240–I3600) was close to 1 within 40 days after wheat emergence, but it was generally less than 1 after 40 days (heading stage). Notably, the NNI of N270 treatments (N270–I4500, N270–I4200, N270–I3900, N270–I3600) showed nitrogen surplus in the early wheat growth stage, with a value close to 1 during the whole growth period, indicating an optimal nitrogen nutritional status. In most cases, the NNI of N300 treatments was the highest and generally greater than 1, showing excess nitrogen (except that N300–I4500 and N270–I4200 were close to 1). Although adequate soil moisture promotes the horizontal and vertical migration of nitrogen in the soil, excess water supply increases the risk of nitrogen leaching to deep soil, especially nitrate nitrogen, thereby limiting wheat nitrogen uptake (Wan et al., 2018). Thus, in this experiment, NNI under different irrigation levels was in the following order: I4500< I4200< I3900< I3600.

**Figure 7:**
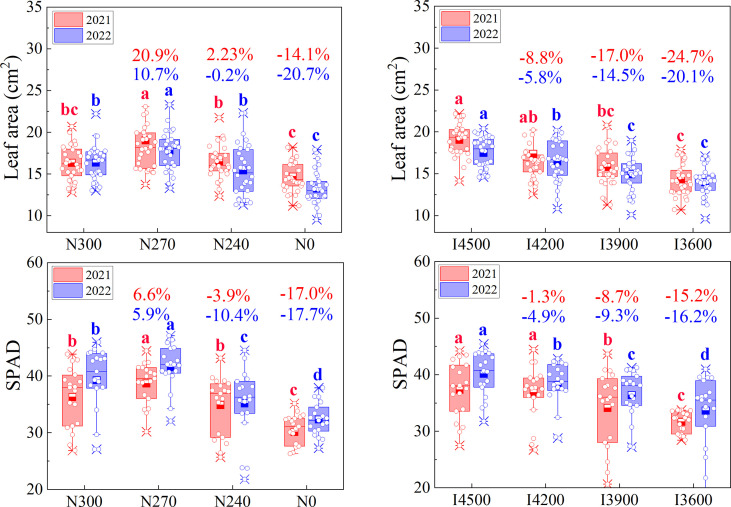
Effects of irrigation and nitrogen levels on flag leaf area and SPAD at anthesis stage. I4500, I4200, I3900, and I3600 indicate 4500, 4200, 3900, and 3600 m^3^ ha^−1^ irrigation levels, respectively. N300, N270, N240, and N0 indicate 300, 270, 240, and 0 kg ha^−1^ nitrogen levels, respectively. The numbers in the columns indicate the percentage change in flag leaf area and SPAD under a given treatment compared with the traditional irrigation (I4500) and nitrogen (N300) levels. The lowercase letters refer to significant differences among the treatments at the 0.05 level.

The photosynthetic performance of leaves is closely related to the nitrogen status of crops (Li et al., 2009). Appropriate nitrogen increases leaf area, improving photosynthetic electron transfer rate and optimizing material accumulation and yield composition (Zhang et al., 2011). In this experiment, compared with the traditional nitrogen level of N300, the leaf area at anthesis first significantly increased (10.7%–20.9%) in N270(**Figure 7**). In most cases, further reduction in nitrogen level reduced leaf area (except N240 leaf area increased by 2.23% in 2021). The leaf area first increased and then declined with the decline in nitrogen level, shown the linear relationship with the dry matter mass that change in nitrogen level (**Figures 4**, **7**). This observation indicated that the increase in dry matter (N270) after moderate reduction in nitrogen supply might be mainly due to the increase in leaf area. The leaf area decreased with the decline in irrigation, indicating that the drop in water supply is not conducive to leaf growth, consistent with previous reports (Shangguan et al., 2000). In addition, good nitrogen nutrition often results in higher chlorophyll content (Li et al., 2009; Ma, 2009). In this experiment, the SPAD showed a significantly increase and then decline with the decline in nitrogen level, consistent with the trend in leaf area (**Figure 7**). This difference in the wheat canopy was visible under different treatments (**Figure 1**). During the middle and late stages of wheat growth, leaves form the main structure of the canopy and is the primary site for photosynthetic assimilation (Gifford and Evans, 1981; Vicentea et al., 2018). Therefore, increasing leaf area and SPAD is conducive to optimizing wheat material accumulation and redistributed. This well explained that dry matter accumulation and redistributed were also optimized to some extent when nitrogen level declined in this experiment (**Figure 4**). In general, severe lack of nitrogen and water will cause abscisic acid (ABA) in wheat roots. ABA will induce stomatal closure and limit photosynthesis when redistributed to wheat leaves, which will eventually lead to the production of lower photosynthetic assimilates (Chaves et al., 2009). This may be the main reason for lower leaf area, biomass and yield under low nitrogen water treatment. In addition, nitrogen application showed a threshold effect in the regulation of crop growth and yield, i.e. excessive nitrogen application was unfavorable to crop growth and yield (Mon et al., 2016; Wang et al., 2017; Wang et al., 2018). In this experiment, the threshold effect under high nitrogen environment (N300) was directly reflected in the significant reduction of leaf area and SPAD value. This ultimately leads to a decline in biomass and yield. Therefore, appropriate nitrogen application rates may result in optimal leaf area and aboveground biomass, and improved grain yield (Si et al., 2020). In our study, the leaf area, SPAD, aboveground biomass, and grain yield showed rising trends with increasing nitrogen application until a rate of 270 kg ha^−1^, after which wheat growth and grain yield began to decrease. This is consistent with the results of previous studies.

## 4 Conclusions

We explored rational water and nitrogen strategies under TR6S, a new spring wheat planting system in Xinjiang. Compared with the traditional irrigation (4500 m^3^ ha^−1^) and nitrogen (300 kg ha^−1^) strategy, a moderate reduction in the irrigation and nitrogen levels resulted in the highest grain yield, WUE, AEN, and profit in two growing seasons. The main reasons for the increase in yield were as follows: (1) optimization of the yield structure and increase in 1000-grain weight and kernels per spike, (2) maintenance of higher TDA and redistribution of DAR to the grains, (3) maintenance of more suitable nitrogen nutrition and improvement in photosynthetic performance. However, the present study followed the traditional strategy’s water and nitrogen supply ratio. Moreover, the NNI of each treatment reflected nitrogen excess in the early stage but nitrogen deficiency in the late stage. Therefore, on the basis of the irrigation and nitrogen strategy recommended in this experiment, combined with irrigation frequency, especially considering the appropriate delayed nitrogen application may be the focus of our future research.

## Data availability statement

The original contributions presented in the study are included in the article/**Supplementary Material**. Further inquiries can be directed to the corresponding author.

## Author contributions

Conceptualization, WW, YZ, XL, JX, KL, SG, YC, HC, XC, PW, HX, and MD; Data curation, WW; Formal analysis, WW and YZ; Investigation, WW, YZ, XL, JX, KL, SG, YC, HC, XC, and PW; Methodology, WW, YZ, HX, and MD; Resources, HX and MD; Software, WW and YZ; Writing–original draft, WW and YZ; Writing–review and editing, HX and MD. WW and YZ contribute equally to the paper. MD (correspondence author) had the overall responsibility for experimental design, project management and manuscript preparation. All authors contributed to the article and approved the submitted version.

## Funding

This study was supported by the projects of Shihezi University Innovation and Development Special Project (CXFZ202014).

## Acknowledgments

We are grateful to XL (Shandong Agricultural University) and Dr. Yunshan Yang (Shihezi University) for the help with manuscript writing. We also thank the reviewers for helping us improve our manuscript. 

## Conflict of interest

The authors declare that the research was conducted in the absence of any commercial or financial relationships that could be construed as a potential conflict of interest.

## Publisher’s note

All claims expressed in this article are solely those of the authors and do not necessarily represent those of their affiliated organizations, or those of the publisher, the editors and the reviewers. Any product that may be evaluated in this article, or claim that may be made by its manufacturer, is not guaranteed or endorsed by the publisher.
