# Supplementary figures and images for: A moderate reduction in irrigation and nitrogen improves water-nitrogen use efficiency, productivity, and profit under new type of drip irrigated spring wheat system
Source: Front Plant Sci. 2022 Oct 10;13:1005945. doi: 10.3389/fpls.2022.1005945 (PMC9589231; doi:10.3389/fpls.2022.1005945)

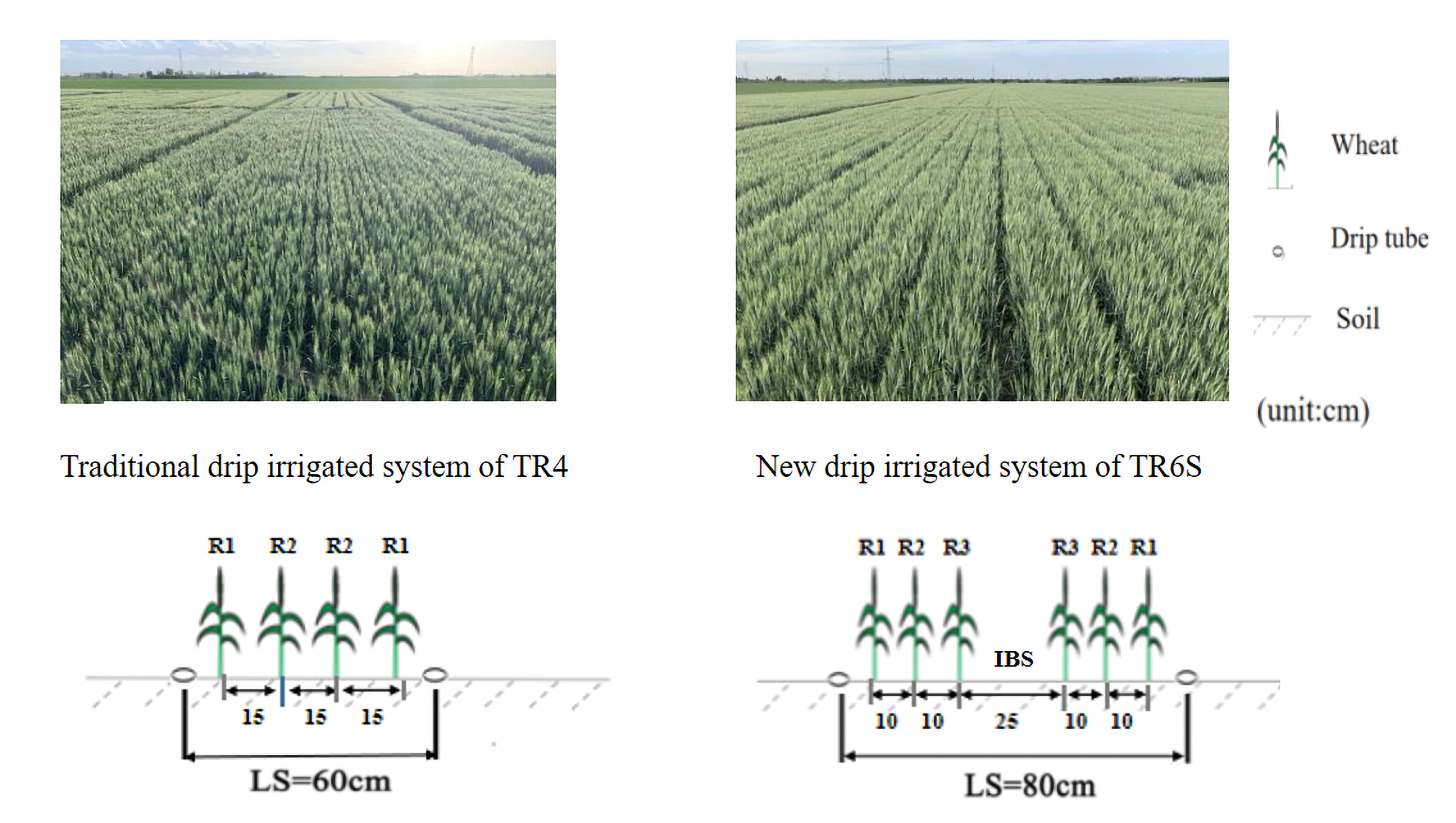

Supplement: Supplementary file 1 [file Image_1.tif]

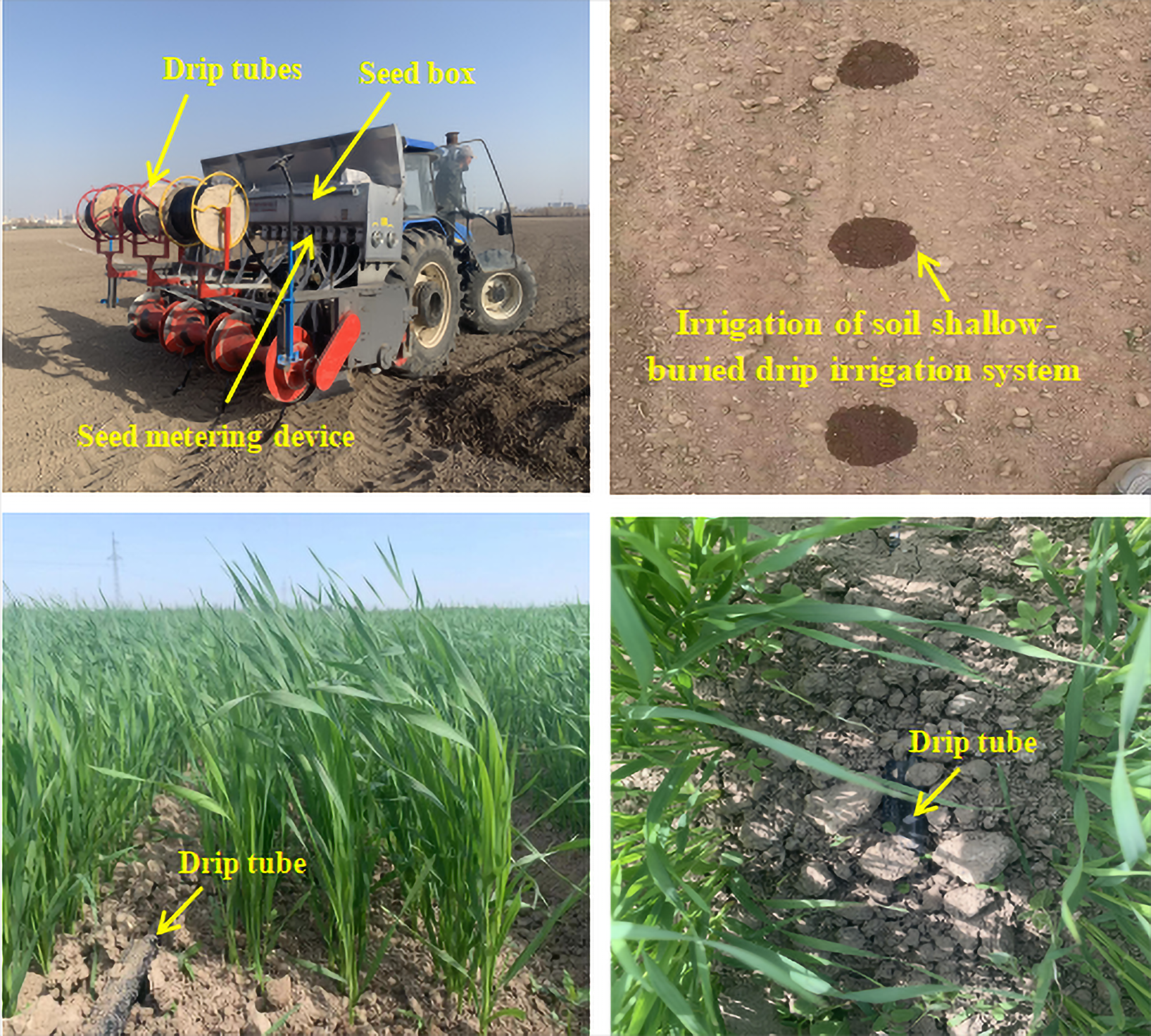

Supplement: Supplementary file 2 [file Image_2.tif]
